# Association Between Inpatient Medication Treatment for Opioid Use Disorder and Reduced One-Year All-Cause Mortality in Patients With Invasive Bacterial Infections

**DOI:** 10.1093/ofid/ofaf061

**Published:** 2025-02-03

**Authors:** Nicholas J Blair, Adam Kopp, Christine Kubin, Jesse Cotton, Michael T Yin, Matthew Scherer

**Affiliations:** Division of Infectious Diseases, Columbia University, New York, New York, USA; Division of Internal Medicine, New York–Presbyterian Hospital, New York, New York, USA; Division of Infectious Diseases, Columbia University, New York, New York, USA; Division of Internal Medicine, New York–Presbyterian Hospital, New York, New York, USA; Division of Infectious Diseases, Columbia University, New York, New York, USA; Division of Internal Medicine, New York–Presbyterian Hospital, New York, New York, USA; College of Physicians and Surgeons, Columbia University, New York, New York, USA; Division of Infectious Diseases, Columbia University, New York, New York, USA; Division of Internal Medicine, New York–Presbyterian Hospital, New York, New York, USA; Division of Infectious Diseases, Columbia University, New York, New York, USA; Division of Internal Medicine, New York–Presbyterian Hospital, New York, New York, USA

**Keywords:** endocarditis, injection drug use, *Staphylococcus aureus*, substance use disorder, buprenorphine, methadone

## Abstract

Invasive bacterial infections are frequent causes of hospitalization among people who use opioids. We evaluated the association between inpatient administration of medication for opioid use disorder with one-year all-cause mortality in patients hospitalized with invasive bacterial infections.

## Graphical Abstract

**Graphical Abstract:**
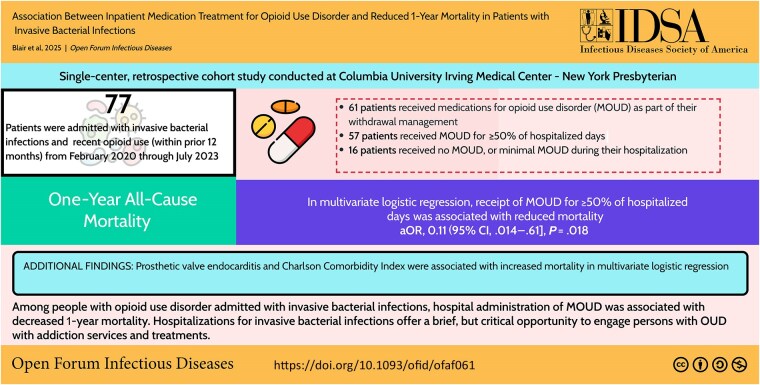
This graphical abstract is also available at Tidbit: https://tidbitapp.io/tidbits/association-between-inpatient-medication-treatment-for-opioid-use-disorder-and-reduced-one-year-mortality-in-patients-with-invasive-bacterial-infections-cfc404a4-891c-4c42-8682-a1c94e82efc3?utm_campaign=tidbitlinkshare&amp;utm_source=IO

Invasive bacterial infections including endocarditis are highly morbid complications of substance use in people with opioid use disorder (OUD) [1–3]. While oral antibiotics can be effective step-down therapy in selected cases of invasive bacterial infections, the Infectious Diseases Society of America recommends between 2 and 6 weeks of intravenous (IV) antibiotics for most cases of bacteremia, including endocarditis [4–9]. Consequently, patients with OUD and invasive bacterial infections often remain hospitalized to complete standard-of-care treatment, as they often cannot access home infusion services or skilled nursing facilities [10].

As people with OUD are often disengaged from traditional primary care [11], these prolonged hospital admissions present a critical opportunity to engage them in medical and mental healthcare and link to outpatient services. Administration of medications for opioid use disorder (MOUD) has been associated with decreased mortality in the outpatient setting [12]. However, rates of linkage to outpatient MOUD from hospitalization remain low in this population [13, 14] and, although some guidelines recommend initiation of MOUD in the inpatient setting [15, 16], the long-term impact on mortality of this practice remains unclear. While prior studies have demonstrated associations between inpatient MOUD and secondary clinical outcomes (reduced patient-directed discharge and readmission rate, increased antibiotic adherence), no study to date has shown a beneficial association between inpatient use of MOUD and mortality [17–20].

In this retrospective analysis we evaluated the association between inpatient administration of MOUD with one-year all-cause mortality in adults with recent opioid use hospitalized with invasive bacterial infections.

## METHODS

We reviewed index admissions for patients with recent opioid use who were hospitalized with bacterial endocarditis between February 2020 and July 2023, as well as all invasive bacterial infections from July 2022 to July 2023, at Columbia University Irving Medical Center (CUIMC). Patients with a history of opioid use, identified through OUD-associated diagnostic codes [21], were screened for inclusion if they were hospitalized during the study period. All hospitalizations associated with OUD and endocarditis diagnostic codes (I33.0, I33.9, I38) between February 2020 and July 2023 were reviewed. Additionally, we expanded the review to include all hospitalizations for other bloodstream infections from July 2022 through July 2023, after the introduction of a new rapid diagnostic test, the Blood Culture Identification Detection Panel 2 (BioFire Diagnostics, bioMérieux, Salt Lake City, Utah), in July 2022.

After the initial screening, patients’ medical records were reviewed in detail to confirm both substance use history and active infection. This study uses data from a larger dataset in which data on invasive infections in people with a wide range of substance use disorders were included, but for the purposes of this analysis, participants were only included if they had active opioid use. Subjects were included if they (1) were aged 18–65 years, (2) had a positive blood culture with an organism known to cause invasive disease, and (3) had documented opioid use within the previous year. We included patients who used drugs by noninjection routes (intranasal, inhalation).

Diagnosis of endocarditis was determined by chart review using updated 2023 Duke criteria [22]; any case meeting criteria for “definite” or “possible” endocarditis was defined as endocarditis in this study, unless otherwise noted by the infectious diseases consult team during admission. The infection type reflects the most significant complication of the patient's invasive bacterial infection (eg, endocarditis, osteomyelitis, septic arthritis). Patients were considered to have completed antibiotics if they finished the recommended duration of treatment, regardless of treatment strategy (eg, full IV vs partial oral or dalbavancin).

Opioid and other substance use history was determined by chart review after initial diagnostic code screening. This review included urine toxicology, patient report, and physical examination findings. We excluded patients in whom opioid use was not documented in any notes or in urine toxicology within the year prior to admission. We did not use the *Diagnostic and Statistical Manual of Mental Disorders, Fifth Edition* (DSM-V) criteria to adjudicate a formal substance use diagnosis, that is, OUD. Administration of MOUD (buprenorphine, methadone, or naltrexone) was operationalized as a treatment exposure if received for ≥50% of hospital days, therefore excluding patients who received minimal or nontherapeutic doses (eg, 1 dose of methadone in the emergency department). At our institution, the psychiatry consult service manages inpatient withdrawal and buprenorphine as we do not have a dedicated addiction medicine team. Primary teams can also prescribe MOUD at our institution, but the psychiatry team would take on MOUD management if consulted.

The definition of housing instability varies in the literature [23]. For the purposes of this study, we used the definition provided by the Centers for Disease Control and Prevention, which has been used in a large study of housing instability in persons with human immunodeficiency virus [24]. Patients were considered stably housed if they were domiciled in an apartment, house, or nursing home. Unstable housing was defined as street homelessness, residence in the shelter system, or transitional housing such as a single-room occupancy. Race and ethnicity are self-reported in the electronic medical record and were limited to the categories in Table 1.

**Table 1.** Baseline Characteristics of Hospitalized Patients With Opioid Use Disorder and Invasive Bacterial Infections (N = 77)

Characteristic	All Patients	MOUD <50%	MOUD ≥50%	*P* Value
	(n = 77)	(n = 20)	(n = 57)	
Age, y, median (IQR)	40 (35–45)	44 (40–53)	37 (34–44)	.006
Female	25 (32)	5 (25)	20 (35)	
Unstable housing	41 (53)	9 (45)	32 (56)	
Race/ethnicity				
Non-Hispanic Black	10 (13)	2 (10)	8 (14)	
Non-Hispanic White	26 (34)	5 (25)	21 (37)	
Hispanic	29 (38)	9 (45)	20 (35)	
Other	12 (16)	4 (20)	8 (14)	
Substance use characteristics				
Injection drug use (≤12 mo)	68 (88)	14 (70)	54 (95)	.008
Frequency of use				
Daily	51 (66)	6 (30)	45 (79)	
Weekly	5 (7)	2 (10)	3 (5)	
Not reported	21 (27)	12 (60)	9 (16)	
Last use				
≤72 h	50 (65)	10 (50)	40 (70)	
≤1 wk	10 (13)	2 (10)	8 (14)	
≤3 mo	6 (8)	2 (10)	4 (7)	
Cocaine use	51 (66)	13 (65)	38 (67)	
Methamphetamine use	10 (13)	3 (15)	7 (12)	
Any stimulant use	54 (70)	14 (70)	40 (70)	
Alcohol use	11 (14)	5 (25)	6 (11)	
MOUD history				
Prior MOUD (ever)	52 (68)	10 (50)	42 (74)	
On MOUD at admission	24 (31)	2 (10)	22 (39)	.018
Buprenorphine	4 (5)	0 (0)	4 (7)	
Methadone	20 (26)	2 (10)	18 (32)	
Medical comorbidities				
Charlson Comorbidity Index	1 (1–2)	1 (1–3)	1 (1–2)	
HIV	10 (13)	4 (20)	6 (11)	
Hepatitis C (any history)	59 (77)	11 (55)	48 (84)	.013
Infection				
Native valve endocarditis	38 (49)	8 (40)	30 (53)	
Prosthetic valve endocarditis	5 (7)	1 (5)	4 (7)	
Bacteremia only	9 (12)	4 (21)	5 (9)	
Cutaneous SSTI or abscess	8 (10)	1 (5)	7 (12)	
Vertebral osteomyelitis	6 (8)	0 (0)	6 (10)	
Nonvertebral osteomyelitis	2 (3)	0 (0)	2 (3)	
Pulmonary	2 (3)	1 (5)	1 (2)	
Septic arthritis	4 (5)	2 (10)	2 (3)	
Other	3 (4)	2 (11)	1 (2)	
Microbiology				
MRSA	31 (40)	7 (35)	24 (42)	
MSSA	22 (29)	3 (15)	19 (33)	
*Streptococcus* spp.	17 (22)	6 (30)	11 (19)	
Fungal	1 (1)	0 (0)	1 (2)	
*Enterococcus*	1 (1)	1 (5)	0 (0)	
Gram-negative bacilli	5 (7)	3 (15)	2 (4)	
*Staphylococcus aureus*	53 (69)	10 (50)	43 (75)	.035
Polymicrobial	10 (13)	2 (10)	8 (14)	
Treatment exposure				
Infectious diseases consultation	74 (96)	18 (90)	56 (98)	
Days of antibiotics received, median (IQR)	28 (12–42)	18 (13–40)	34 (10–42)	
Psychiatry consultation	57 (74)	12 (60)	45 (79)	
Inpatient MOUD treatment				
No administration of MOUD	14 (18)	14 (70)	0 (0)	
Started and discontinued	2 (3)	2 (10)	0 (0)	
Methadone only	44 (57)	3 (15)	41 (72)	
Methadone to buprenorphine	8 (10)	0 (4)	8 (14)	
Buprenorphine only	8 (10)	1 (5)	7 (12)	
Naltrexone	1 (1)	0 (0)	1 (2)	
Adjunct medications	58 (74)	11 (58)	47 (81)	
Outcomes				
LOS, days, median (IQR)	23 (8–50)	16 (7–29)	26 (10–52)	
Completed antibiotics	58 (75)	17 (85)	41 (72)	
Patient-directed discharge	34 (44)	8 (40)	26 (46)	
1-y all-cause mortality	10 (13)	6 (30)	4 (7)	.016
Confirmed outcome^a^	61 (79)	15 (75)	46 (81)	

Data are presented as No. (%) unless otherwise indicated.

Abbreviations: HIV, human immunodeficiency virus; IQR, interquartile range; LOS, length of stay; MOUD, medications for opioid use disorder; MRSA, methicillin-resistant *Staphylococcus aureus*; MSSA, methicillin-sensitive *Staphylococcus aureus*; SSTI, skin or soft tissue infection.

^a^Able to confirm outcome within the available medical record.

The primary outcome of interest was one-year all-cause mortality, inclusive of in-hospital deaths. In addition, patients who survived their index admission were followed up to 1 year after leaving the hospital within the available medical record. Mortality was adjudicated based on review of our systemwide medical record (Epic), which links with the Social Security death registry.

### Statistical Analysis

We collected patient demographics, microbiology, and medical and substance use history up to 1 year after the index admission. All analyses were performed using R Statistical Software (R version 4.2.3, R Core Team 2023). *P* values were derived from 2-sample *t* test or Wilcoxon rank-sum test for continuous variables and χ^2^ or Fisher exact test for categorical variables when appropriate. Univariate and multivariate logistic regression models were used to evaluate the risk of 1-year mortality using odds ratios (ORs). Only variables with *P* < .10 in the univariate model were included in the final multivariate model.

### Patient Consent

This study was approved and granted exemption by the Institutional Review Board of CUIMC (IRB-AAAV2011). Therefore, individual patient consent was not required for the study.

## RESULTS

During the study period, we identified 77 patients who met criteria for infection and opioid use within 12 months of admission. For patients with multiple hospitalizations, the index admission was analyzed. Baseline demographics, index infection characteristics, and outcome data of interest are displayed in Table 1. MOUD and no-MOUD groups did not differ significantly in terms of race, gender, housing status, infection type, or presence of concurrent stimulant use. There were similar rates of psychiatry consultation between the 2 groups. The MOUD group was younger, had higher rates of injection drug use and hepatitis C, a higher proportion of *Staphylococcus aureus* infections, and a longer recommended duration of antibiotic therapy. Inpatient MOUD strategy can be found in Table 1. The MOUD group displayed lower 1-year mortality than the no-MOUD group in direct comparison (6/20 [30%] vs 4/57 [7%], *P* = .016). Completion of standard-of-care IV antibiotics was low (25/77 [32%] overall) and did not differ significantly between groups.

In multivariate logistic regression, hospital administration of MOUD was significantly associated with reduced 1-year mortality (adjusted OR [aOR], 0.11 [95% confidence interval {CI}, .014–.61]; *P* = .018) after adjusting for prosthetic valve endocarditis (aOR, 46.8 [95% CI, 4.67–727]; *P* = .002) and Charlson Comorbidity Index (aOR, 1.36 [95% CI, 1.03–1.83]; *P* = .031), which were the only other variables that had also demonstrated statistically significant associations with 1-year mortality in the univariate regression model (Table 2). There were 4 patients who received MOUD as part of their treatment strategy for less than half of hospitalized days. All 4 of these patients started MOUD in the latter half of their hospitalizations (3 with methadone, 1 with buprenorphine). We performed a sensitivity analysis in which 4 patients who started MOUD in the latter part of their hospitalization (therefore receiving MOUD for <50% of hospital days) were reclassified into the MOUD group. This produced similar results when regression models were rerun (Supplementary Table 1).

**Table 2.** One-Year All-Cause Mortality of Patients With Opioid Use Disorder and Invasive Bacterial Infections (N = 77)

Characteristic	Univariate Model		Multivariate Model	
	OR (95% CI)	*P* Value^a^	aOR (95% CI)	*P* Value^b^
Age	1.02 (.95–1.09)	.53	…	
Gender (female)	0.88 (.18–3.49)	.86	…	
Housing (stable)	1.85 (.48–7.81)	.37	…	
Any stimulant use	0.99 (.25–4.96)	.99	…	
Cocaine use	1.22 (.31–6.07)	.79	…	
Methamphetamine use	0.72 (.037–4.54)	.76	…	
Alcohol	0.63 (.033–3.96)	.68	…	
Last drug use (within 72 h)	0.49 (.12–1.93)	.3	…	
Injection drug use (within 12 mo)	0.47 (.092–3.49)	.39	…	
On MOUD at admission	0.94 (.19–3.75)	.93	…	
Charlson Comorbidity Index	1.28 (1.01–1.62)	.**036**	1.36 (1.03–1.83)	.**031**
HIV	3.67 (.68–17.0)	.1	…	
Infection				
Prosthetic valve endocarditis	13.9 (2.0–121)	.**0082**	46.8 (4.67–727)	.**002**
Native valve endocarditis	1.03 (.26–4.02)	.97	…	
*Staphylococcus aureus*	1.96 (.44–13.7)	.42	…	
Treatment exposure				
Psychiatry consultation	1.47 (.33–10.3)	.65	…	
Received MOUD^c^	0.18 (.040–.70)	.**015**	0.11 (.014–.61)	.**018**
Received adjunct medications	0.43 (.11–1.88)	.24	…	

*P*-values in bold if < 0.1.

Abbreviations: aOR, adjusted odds ratio; CI, confidence interval; HIV, human immunodeficiency virus; MOUD, medications for opioid use disorder; OR, odds ratio.

^a^ *P* value from univariate logistic regression.

^b^ *P* value from multivariate logistic regression.

^c^Patients who received MOUD ≥50% of hospitalized days (n = 57).

Half (3/6 [50%]) of patients who died in the hospital had left-sided endocarditis and were not offered valve replacement (Supplementary Table 2). In all 3 cases, the patients were deemed nonsurgical candidates due to past substance use relapses and perceived inability to engage in care for their substance use disorder.

## DISCUSSION

In this retrospective study, inpatient administration of MOUD was associated with decreased all-cause 1-year mortality in people with OUD admitted with invasive bacterial infections. To our knowledge, this is the first report of a mortality benefit associated with receipt of MOUD during an infection-related hospitalization. These findings contribute to a growing body of evidence highlighting the benefits of MOUD during hospitalizations for invasive bacterial infections.

Our findings are particularly noteworthy as the MOUD group exhibited a higher incidence of *S aureus* infections and a prevalence of infective endocarditis exceeding 50%, factors that might be expected to be associated with higher mortality rates. One potential mechanism for the reduced mortality observed in the MOUD group, despite the clinical differences, is the longer duration of antibiotic therapy (34 days compared to 18 days). Although this disparity was not statistically significant in our study—likely due to the small sample size—it may have contributed to lower mortality despite the greater prevalence of *S aureus* and endocarditis. Previous studies have shown that initiating MOUD during hospitalization is associated with increased use of gold-standard IV antibiotic therapy [25], reduced occurrence of patient-directed discharges [19, 20], fewer readmissions [17, 18], shorter length of stay [26], and improved linkage to outpatient care [27]. Despite a robust evidence base for initiating MOUD during hospitalization for infection, many hospitalized patients with OUD do not receive treatment at all, and linkage to outpatient services remains suboptimal [19, 20].

The reasons behind our current state of practice are multifactorial, including both provider and systemic barriers [27]. On the provider level, many physicians and advanced care practitioners lack training in MOUD prescription, which may lead to discomfort in managing severe opioid withdrawal and prescribing buprenorphine upon discharge or for outpatient care [28]. On a systemic level, the availability of outpatient MOUD access points are not equally distributed. Opioid treatment programs prescribing methadone are more prevalent in majority African American and Hispanic communities, whereas office-based opioid treatment using buprenorphine are more common in majority White communities [21, 29]. While we did not observe any racial disparities regarding inpatient MOUD strategies, the higher rates of inpatient methadone initiation versus buprenorphine initiation in this cohort of predominantly non-White patients might reflect inpatient providers’ awareness of the relative lack of availability of outpatient buprenorphine in this community.

Of note, 60% (6/10) of patient deaths occurred during the index admission, and 80% (8/10) of deaths occurred due to infectious complications, which demonstrates both the acuity of injection-related infections and the importance of addressing OUD treatment early in the hospitalization. Three people who use drugs were not offered surgical intervention, potentially life-saving, in part because surgical teams may be hesitant to operate if there is no addiction treatment plan in place. Historically, there have been poor long-term outcomes in patients who undergo valve replacement with concurrent substance use [30], and cardiothoracic surgeons are less likely to operate in cases of injection-related endocarditis [31]. To address these concerns about long-term follow-up, many hospitals have implemented transitional programs in order to meet the needs of this patient population [32].

The study has several limitations, including its nonrandomized single-site design and relatively small sample size. As this was a nonrandomized retrospective study, it is also possible that mortality and receipt of MOUD are related in a co-linear fashion (ie, healthier patients are preferentially being administered MOUD) rather than MOUD itself being protective against mortality. We opted to focus on infective endocarditis and other bloodstream infections due to the ease of identifying these cases in the electronic medical record; however, bloodstream infections represent only a fraction of all infections among OUD. In addition, we likely missed many bloodstream infections in the earlier period of the study before the introduction of the Blood Culture Identification Detection Panel 2.

## CONCLUSIONS

Among people who use opioids admitted with invasive bacterial infections, hospital administration of MOUD was associated with decreased 1-year mortality. Hospitalizations for invasive bacterial infections offer a brief but critical opportunity to engage people who use opioids with addiction services and treatments.

## Supplementary Material

ofaf061_Supplementary_Data

## Supplementary Data

Supplementary materials are available at *Open Forum Infectious Diseases* online. Consisting of data provided by the authors to benefit the reader, the posted materials are not copyedited and are the sole responsibility of the authors, so questions or comments should be addressed to the corresponding author.

## Notes

***Author contributions.*** All authors contributed significantly to this work. N. J. B., M. S., and M. T. Y. conceived the study, collected and analyzed the data, and prepared the manuscript. A. K., C. K., and J. C. were instrumental in data abstraction. All authors were involved in writing of the manuscript.

***Acknowledgments.*** We would like to thank the CUIMC Department of Infectious Diseases for supporting our work including the many dedicated physicians, nurses, and other staff who are dedicated to the care of people with substance use disorders.

***Financial support.*** This work was unfunded but was supported by the CUIMC Department of Infectious Diseases.
