# Supplementary material for: Association Between Inpatient Medication Treatment for Opioid Use Disorder and Reduced One-Year All-Cause Mortality in Patients With Invasive Bacterial Infections
Source: Open Forum Infect Dis. 2025 Feb 3;12(3):ofaf061. doi: 10.1093/ofid/ofaf061 (PMC11886840; doi:10.1093/ofid/ofaf061)
Supplement: ofaf061_Supplementary_Data [file ofaf061_supplementary_data.docx]

| **Supplemental Table 1:** Sensitivity analysis of one-year all-cause mortality of patients with opioid use disorder and invasive bacterial infections (N= 77) | | | | |
| --- | --- | --- | --- | --- |
|  | Univariate Model | | Multivariate Model | |
|  | OR (95% CI) | P-value* | aOR (95% CI) | P-value** |
| Age | 1.02 (0.95 - 1.09) | 0.53 |  |  |
| Gender (female) | 0.88 (0.18 - 3.49) | 0.86 |  |  |
| Housing (stable) | 1.85 (0.48 - 7.81) | 0.37 |  |  |
| Any stimulant use | 0.99 (0.25 - 4.96) | 0.99 |  |  |
| Cocaine Use | 1.22 (0.31 - 6.07) | 0.79 |  |  |
| Methamphetamine Use | 0.72 (0.037 - 4.54) | 0.76 |  |  |
| Alcohol | 0.63 (0.033 - 3.96) | 0.68 |  |  |
| Last drug use (within 72 hours) | 0.49 (0.12 - 1.93) | 0.3 |  |  |
| Injection drug use (within 12 months) | 0.47 (0.092 - 3.49) | 0.39 |  |  |
| On MOUD at admit | 0.94 (0.19 - 3.75) | 0.93 |  |  |
| Charleson comorbidity index | 1.28 (1.01 - 1.62) | **0.036** | 1.33 (1.02 - 1.76) | **0.034** |
| HIV | 3.67 (0.68 - 17.0) | 0.1 |  |  |
| Infection |  |  |  |  |
| Prosthetic valve endocarditis | 13.9 (2.0 - 121) | **0.0082** | 31.5 (3.61 - 374) | **0.0025** |
| Native valve endocarditis | 1.03 (0.26 - 4.02) | 0.97 |  |  |
| *Staphylococcus aureus* | 1.96 (0.44 - 13.7) | 0.42 |  |  |
| Treatment exposure |  |  |  |  |
| Psychiatry Consult | 1.47 (0.33 - 10.3) | 0.65 |  |  |
| Received MOUD^ | 0.20 (0.047 - 0.81) | **0.023** | 0.17 (0.029 - 0.92) | **0.041** |
| Received adjunct medications | 0.43 (0.11 - 1.88) | 0.24 |  |  |
| **P-values in bold if < 0.1** | | | | |
| Abbreviations: MOUD = medications for opioid use disorder | | | | |
| ^ Four patients who received MOUD as part of their treatment plan, but for < 50% hospitalized days. For this regression they were reclassified to the “received MOUD” group. After this reclassification, 61 patients received MOUD and 16 received none or minimal MOUD. | | | | |
|  | | | | |
|  | | | | |
|  | | | | |

| **Supplemental Table 2:** Causes of one-year all-cause mortality of patients with opioid use disorder and invasive bacterial infections (N = 10) | | | | | | | |
| --- | --- | --- | --- | --- | --- | --- | --- |
| Patient | Age | CCI | Details | Location | Infection-Related | Pre-admit  MOUD | Admission MOUD |
| 1 | 30 | 1 | Underwent valve surgery for MRSA PVE and died in early post-operative period from cardiac arrest. | CUIMC, index admission | Unclear | Yes, buprenorphine | Yes, buprenorphine |
| 2 | 31 | 0 | MSSA PVE complicated by PDD. Died from recurrent PVE at an outside hospital. | Post-discharge | Yes | Yes, methadone | Yes, methadone |
| 3 | 34 | 1 | MRSA bacteremia with residual pulmonary disease. Discharged on methadone. Died from MSSA bacteremia on separate admission. | Post-discharge | Yes | No | Yes, methadone |
| 4 | 42 | 1 | *S. mitis* aortic and mitral valve endocarditis. Prescribed high dose amoxicillin after valve surgery and PDD. Died during readmission for polymicrobial PVE | Post-discharge | Yes | No | No |
| 5 | 44 | 7 | MSSA aortic and native mitral valve endocarditis. Ethics team consulted and surgery was not offered. | CUIMC, index admission | Yes | No | No |
| 6 | 44 | 8 | Found on park bench, unknown cause. Confirmed by clinic social worker. | Post-discharge | Unknown | No | No |
| 7 | 46 | 2 | Persistent MRSA bacteremia with multiple intraabdominal abscesses. | CUIMC, index admission | Yes | No | Yes, Methadone |
| 8 | 49 | 1 | *S. parasanguinous* prosthetic aortic and mitral valve endocarditis. Ethics team consulted and surgery was not offered. Died in hospital from complications of infection. | CUIMC, index admission | Yes | No | No |
| 9 | 51 | 2 | MSSA mitral valve endocarditis. Surgery was not offered. Taking methadone at time of admission | CUIMC, index admission | Yes | Yes, methadone | Yes, methadone |
| 10 | 62 | 10 | MRSA bacteremia from skin source, course complicated by aspiration pneumonia | CUIMC, index admission | Yes | No | No |

**Abbreviations:** CCI, Charleson comorbidity index; MRSA, methicillin-resistant *Staphylococcus aureus*; PVE, prosthetic valve endocarditis; CUIMC, Columbia University Irving Medical Center; PDD, patient directed discharge; MSSA, methicillin-sensitive *Staphylococcus aureus;* MOUD, medication for opioid use disorder
